# Identifying Risk and Resilience Factors in the Intergenerational Cycle of Maltreatment: Results From the *TRANS-GEN* Study Investigating the Effects of Maternal Attachment and Social Support on Child Attachment and Cardiovascular Stress Physiology

**DOI:** 10.3389/fnhum.2022.890262

**Published:** 2022-07-18

**Authors:** Anna Buchheim, Ute Ziegenhain, Heinz Kindler, Christiane Waller, Harald Gündel, Alexander Karabatsiakis, Jörg Fegert

**Affiliations:** ^1^Department of Clinical Psychology II, Institute of Psychology, University of Innsbruck, Innsbruck, Austria; ^2^Department of Child and Adolescent Psychiatry and Psychotherapy, Ulm University Hospital, Ulm, Germany; ^3^German Youth Institute, Munich, Germany; ^4^Department of Psychosomatic Medicine and Psychotherapy, Nuremberg General Hospital, Paracelsus Medical University, Nürnberg, Germany; ^5^Department of Psychosomatic Medicine and Psychotherapy, Ulm University, Ulm, Germany

**Keywords:** childhood maltreatment, intergenerational cycle of attachment, attachment representation, social support, maternal sensitivity, adult attachment projective picture system, oxytocin receptor gene, respiratory sinus arrhythmia

## Abstract

**Introduction:**

Childhood maltreatment (CM) is a developmental risk factor and can negatively influence later psychological functioning, health, and development in the next generation. A comprehensive understanding of the biopsychosocial underpinnings of CM transmission would allow to identify protective factors that could disrupt the intergenerational CM risk cycle. This study examined the consequences of maternal CM and the effects of psychosocial and biological resilience factors on child attachment and stress-regulatory development using a prospective *trans*-disciplinary approach.

**Methods:**

Mother-child dyads (*N* = 158) participated shortly after parturition (*t*_0_), after 3 months (*t*_1_), and 12 months later (*t*_2_). Mothers’ CM experiences were assessed at *t*_0_, attachment representation at *t*_1_ and psychosocial risk and social support were assessed at *t*_1_ and *t*_2_. At *t*_2_, dyads participated in the Strange Situation Procedure (SSP). Children’s attachmen status were classified as organized vs. disorganized, including their level of disorganized behavior, and heart rate (HR) and respiratory sinus arrhythmia (RSA) were recorded as stress response measures of the autonomic nervous system. Maternal caregiving during SSP was assessed using the AMBIANCE scale. Child’s single nucleotide polymorphisms rs*2254298* within the oxytocin receptor (OXTR) and rs*2740210* of the oxytocin gene *(OXT)* were genotyped using DNA isolated from cord blood.

**Results:**

Maternal CM experiences (CM+) were significantly associated with an unresolved attachment status, higher perceived stress and more psychological symptoms. These negative effects of CM were attenuated by social support. As expected, maternal unresolved attachment and child disorganized attachment were significantly associated. Maternal caregiving did not mediate the relationship between maternal and child attachment but influenced children’s HR and RSA response and disorganized behavior. Moreover, the rs*2254298* genotype of the OXTR gene moderated the stress response of children from mothers with CM. Children carrying the rs*2740210* risk allele of the OXT gene showed more disorganized behavior independent from maternal CM experiences.

**Conclusion:**

We replicated and extended existing CM and attachment models by co-examining maternal attachment, social support, and child genetic susceptibility on child attachment and cardiovascular stress regulation. The findings contribute to an extended understanding of risk and resilience factors and enable professionals to target adequate services to parents and children at risk.

## Introduction

Childhood maltreatment (CM) includes sexual, physical, emotional abuse, and physical and emotional neglect and is considered a repetitive and chronic stressor rather than a single event (Madigan et al., 2019). CM can impair the psychological and biological development of affected individuals and also their later functioning in adolescence and adulthood, especially when it occurs early in life (Jaffee and Maikovich-Fong, 2011; Alink et al., 2012; Norman et al., 2012; Gonzalez, 2013; Madigan et al., 2015). The negative consequences of CM on physical and mental health are robustly documented in the literature (for a meta-analysis see, Humphreys et al., 2020), with additional evidence for transmission of adversity and health consequences also to the next generation (Norman et al., 2012; Leve et al., 2015; Narayan et al., 2021).

Research demonstrated that parents with a history of CM are at risk of maltreating their children, most frequently in infancy (Fegert et al., 2010). This finding is termed the *intergenerational cycle of maltreatment* (ICM, Zuravin et al., 1996). However, transmission rates of CM within the first year of the child’s life were reported to vary between 7 to 23% (Pears and Capaldi, 2001; Dixon et al., 2005; Berlin et al., 2011), demonstrating that a substantial, yet varying number of parents with a history of CM become perpetrators. This large variance indicates possible methodological problems of retrospective studies in identifying relevant risk and resilience factors for ICM (Dixon et al., 2009; Masten and Monn, 2015; Woods-Jaeger et al., 2018; Narayan et al., 2021). As a consequence, subsequent approaches examining relevant transmission factors require a prospective research design and the implementation of *trans*-disciplinary methods.

The study *TRANS-GEN* was conceptualized to address this problem using a prospective design covering the first year of mother-child dyads. We focused on the role of the *attachment system*, the *social support system* and *biological stress susceptibility* in mother-child dyads, as these systems are known to affect the consequences of CM to the next generation (Brunner et al., 2015). A better understanding of the biopsychosocial underpinnings of ICM would allow for the identification of *protective* factors that could help to disrupt intergenerational effects. Therefore, the present study aimed at examining the consequences of maternal CM exposure and the buffering effects of maternal psychosocial (attachment status, social support, perceived stress, and psychological symptoms) and child biological resilience factors (OXTR rs2254298 and OXT rs2740210 genotype) on children’s attachment and cardiovascular stress regulation. First, we summarize major findings from previous studies that address the relevant transmission systems to provide the rationale for the given hypotheses and our expanded ICM model.

### Intergenerational Cycle of Maltreatment and Parenting Behavior

Parents with a history of CM may exhibit an elevated risk for impaired parenting (Lang et al., 2010), however, as noted above, not every parent with CM experiences will maltreat their children (Pears and Capaldi, 2001; Madigan et al., 2019). Studies have shown that CM exposure linked to inadequate parenting (e.g., controlling, hostile, disruptive, and unresponsive behavior) has developmental consequences (Lyons-Ruth, 1996; Widom et al., 2015; Kerr and Capaldi, 2019).

The results of a recent review of 29 studies demonstrated significant direct associations between maternal childhood adversities and negative parenting, including physical punishment, inconsistent discipline, emotional maltreatment, emotional abuse, neglect, and coercive and abusive parenting. Factors that play an important role in aggravating these associations include maternal mental health problems, partner violence, and an insecure parent-child relationship (Lotto et al., 2021).

Especially sensitive and responsive caregiving (i.e., accurate interpretation and prompt response to child’s needs, Ainsworth, 1978) can protect children from excessive stress and support the development of effective stress regulation strategies (Laurent et al., 2016). Studies showed that the quality of early parent-child interaction, especially in the child’s first year of life, predicts child behavior outcomes (De Wolff and van IJzendoorn, 1997; Dozier and Bernard, 2017). This finding is especially important in families with a history of CM (Khoury et al., 2021). Lotto et al. (2021) reported that very few studies explored the effects of protective factors that enable breaking the negative cycle of violence across generations. However, other meta-analytic work found maternal insensitivity to be an insufficient antecedent of disorganized attachment, with insensitivity explaining only a small proportion of variance in this outcome (van IJzendoorn et al., 1999; Verhage et al., 2016). A possible explanation for this finding might be that maternal sensitivity measures might capture behaviors falling within a normative range of caregiving, such as sensitively responding to infant cues, but they do not capture the relevant anomalous, disruptive behaviors exhibited by caregivers in disorganized attachment relationships (Lyons-Ruth et al., 1999).

In the present study, we focused on the intergenerational effects of maternal attachment representation, and maternal disruptive behavior on children’s attachment in addition to examining other possible influences on the mother-child dyad.

### Intergenerational Cycle of Maltreatment and Child Attachment

The function of the attachment relationship is to increase the child’s chances of survival (Bowlby, 1973). Attachment is a behavioral system activated by physical or environmental threats and stress (Bowlby, 1973). The quality of attachment in 1-year-old children as measured with the *Strange Situation Procedure* (SSP; Ainsworth, 1978) demonstrates the importance of the caregiver’s ability to be responsive to the child’s physical and psychological needs, especially when stressed. In short, a child’s perceptions of and coping responses to stress are closely linked to the quality of the attachment relationship.

Ainsworth and others observed that separation from the caregiver in the SSP is a mild stressor that elicits a range of responses in 1-year-olds (Main and Solomon, 1986). Their behavioral response patterns can be classified as “secure,” “insecurely organized” (avoidant, ambivalent), and “disorganized” (highly insecure). Secure children have confidence in their caregiver’s availability and flexibly seek comfort as needed. The caregivers of insecurely organized children overly distance themselves or are confused by and preoccupied with their child’s attachment bids. These children manage to get their attachment needs met much of the time; however, their experiences sacrifice developing flexible stress regulation mechanisms (Main, 1990). Children with disorganized attachment are characterized by contradictory and unintegrated behaviors toward the caregiver that interrupt bids for or accept needed proximity or comfort (e.g., freezing, huddling on the floor, and signs of severe approach-avoidance conflict behavior, Main and Solomon, 1986). Classification as disorganized is determined by rating the frequency and intensity of disorganized behavior. Disorganized attachment indicates the risk of an insufficient ability to cope with attachment-related stress. Disorganized attachment is a relevant risk factor for children’s emotional and cognitive development and is associated with internalizing and externalizing behavior (O’Connor et al., 2011; Groh et al., 2017), psychopathology (Liotti, 1992; Jacobvitz et al., 2011; Forslund et al., 2020), and elevated physiological stress responses (see review by Gander and Buchheim, 2015).

In sum, disorganized attachment is considered a developmental risk factor and documented as having a higher incidence in families with CM (Cyr et al., 2010). Therefore, disorganized attachment is an outcome variable of interest in the present ICM study. Our main interest was to study whether children with disorganized attachment are more susceptible to stress and how this response may be related to the behavior of the caregiver.

### Intergenerational Cycle of Maltreatment and Parental Attachment Representation

The parents’ adult attachment system is a major candidate for affecting the intergenerational transmission of CM and attachment to the next generation (van IJzendoorn and Bakermans-Kranenburg, 2019; Zajac et al., 2019). Adult attachment representation is assessed by evaluating narratives of attachment-related themes (Main and Goldwyn, 1998; George and West, 2012). The classification groups are analogous to the groups in children. Secure adults are flexible and thoughtful when describing attachment experiences. Insecure organized adults (dismissing, preoccupied) dismiss the importance of attachment events or are confused by and mentally entangled in caregiver insensitivity. In comparison, adults with an unresolved (disorganized) attachment are flooded and unable to regulate attachment-related trauma like, e.g., abuse, loss or emotional neglect.

Longitudinal studies have demonstrated a robust association between adult attachment representations and child attachment patterns, particularly in parent-infant dyads with maternal unresolved attachment and children with disorganized attachment (Fonagy et al., 1991; Benoit and Parker, 1994; van IJzendoorn, 1995; Steele et al., 1996; Behrens et al., 2016). The rate of unresolved as compared to organized attachments is significantly higher in CM in both clinical and non-clinical groups (Bakermans-Kranenburg and van IJzendoorn, 2009; Bauriedl-Schmidt et al., 2017; Buchheim et al., 2017; Buchheim and Diamond, 2018; Gander et al., 2018, 2020; Gerhardt et al., 2021) and associated with adverse outcomes after experiencing CM.

There is a significant association between maternal unresolved attachment representation and disorganized attachment of children (Schuengel et al., 1999; Madigan et al., 2007; Lyons-Ruth and Jacobvitz, 2016). Mothers with CM engaged in poorer quality interactions with their child and showed a decreased ability to soothe their child’s distress (Lang et al., 2010). Parents with insecure representations, especially parents with unresolved attachment representation, show consistently negative parenting behaviors (Jones et al., 2015). Recently, Zajac and colleagues (Zajac et al., 2019) reported that mothers with dismissing and unresolved attachments, who also experienced CM, showed higher insensitive parenting behavior. These findings support the link between maternal CM experiences, mental representation of their attachment history, and impaired parenting behaviors.

Khan and Renk (2019) suggested to co-examine the association between negative parenting behavior and CM by including maternal insecure attachment as a core influencing factor (Khan and Renk, 2019). Since there is an absence of research that addresses this missing link, this association was of central interest in the present study.

### Intergenerational Cycle of Maltreatment and Stress-Related Effects on Parental Caregiving

Examining the mechanism of intergenerational transmission of attachment, sensitive parenting and children’s attachment security has been a main focus of research for more than four decades. The study findings are mixed, which suggests that sensitivity is not as ubiquitous as expected; transmission discrepancies have led to the concept of a “transmission gap” (van IJzendoorn, 1995; Verhage et al., 2016; van IJzendoorn and Bakermans-Kranenburg, 2019). The gap is especially evident for mothers under stress (van IJzendoorn and Bakermans-Kranenburg, 2019). CM affects subsequent coping, as manifested in long-term stress physiology (MacMillan et al., 2009) and increased health risk behaviors and difficulties (Watts-English et al., 2006). It follows that the transition to parenthood can be challenging particularly for parents struggling with the stress of CM (Deater-Deckard, 1998).

The association between parenting stress and maternal CM and parenting difficulties is well-documented. Studies on ICM have shown that mothers who report more CM and current parenting stress are significantly less sensitive in the interaction with their children (Pereira et al., 2012; Savage et al., 2019; Bérubé et al., 2020). Therefore, this study intended to add new aspects to the literature by identifying possible protective factors that buffer the negative consequences of parental perceived stress and disruptive behavior (e.g., affective communication errors, role/boundary confusion, intrusive behavior, and withdrawal).

### Intergenerational Cycle of Maltreatment and Psychological Distress Risk

Childhood maltreatment is associated with adverse long-term consequences for mental health and psychological symptoms (Humphreys et al., 2020). Recently, Choi et al. (2019) reported that maternal CM was associated with an increased risk for psychopathology including depression. Mothers that suffered from a postpartum (first year after birth) depression had nearly twice the prevalence (40% vs. 22%) of maltreating their children compared to mothers that did not suffer from depression in the postpartum year. These researchers suggested that identifying protective factors and treating maternal psychopathology when their children are young may be an important way to interrupt trauma cycles and improve both maternal and child outcomes. A recent large-scale meta-analysis confirmed this association and highlighted a particularly large effect size between childhood emotional maltreatment and symptoms of psychological distress (Humphreys et al., 2020). This pattern suggests that future ICM studies should identify protective factors that buffer the negative transmission effects of mothers’ CM on psychopathology and impaired parenting.

### Intergenerational Cycle of Maltreatment and Social Support

Social support is one of three resilience factors (in addition to sleep and life satisfaction) buffering the negative effect of adverse childhood experiences on adult physical and mental health (Logan-Greene et al., 2014). Social support is a mediator between CM and adult psychopathology (Runtz and Schallow, 1997; Vranceanu et al., 2007), as CM reduces social support which in turn increases the risk for maternal psychopathology (Vranceanu et al., 2007; Salazar et al., 2011). It follows that high levels of social support may buffer the negative effect of CM. Studies showed that support from family members, partners (father), and social service systems are important protective factors (Afifi and MacMillan, 2011) and enhanced mothers’ self-esteem and self-efficacy, aiding the transition to motherhood, and consequently promoting caregiving and healthy child development (Emmanuel et al., 2011; Fahey and Shenassa, 2013).

Studies also confirmed the protective effect of social support on the association between CM and symptoms of adult post-traumatic stress disorder and depression (Schumm et al., 2006; Salazar et al., 2011; Evans et al., 2013). We propose that mothers with CM are at risk for psychopathology and experiencing higher parental stress levels. Therefore, in the present study, we also examined the attenuating role of social support on the relationship between CM, perceived stress, psychopathology, and maternal impaired parenting. Moreover, we additionally examined the role of institutional support, since most studies focused on social support in ICM samples.

### Intergenerational Cycle of Maltreatment and Child Susceptibility – Stress Regulatory Function

Biological pathways may be the conduit of negative maternal CM effects (Schury and Kolassa, 2012). These are linked to alterations in the development of children’s neuroendocrine (Schury et al., 2017a) and autonomic nervous systems (Roder et al., 2020). However, results from our research consortium *TRANS-GEN* showed that the quality of maternal caregiving during parent–child interaction and not CM *per se* played a crucial role in the regulation of a child’s cortisol stress reactivity after the SSP (Köhler-Dauner et al., 2019a). Children of disruptive mothers showed a significantly higher cortisol increase compared to children of mothers with non-disruptive behavior. Transmission of CM experiences to the next generation appears to be mediated by maternal behavior (Köhler-Dauner et al., 2019a). Another study demonstrated that maternal caregiving influenced the responsiveness of the sympathetic and parasympathetic branches of the autonomic nervous system in children. Heart rate (HR) increased especially when children of disruptive mothers were alone with the stranger during the SSP (Köhler-Dauner et al., 2019b). Both studies highlight the importance of supporting responsive caregiving that enables the children to adapt to stressful attachment-relevant situations.

The functioning of the stress response is suggested to be linked with child attachment (Nachmias et al., 1996; Gander and Buchheim, 2015; Packard et al., 2021). Bernard and Dozier (2010) observed that children with disorganized attachment did not show increased cortisol activity during free play but did show an increase during the mother-separation episode. Children with organized attachments (secure and organized insecure) did not show a cortisol response to either one of the tasks. Also increased cortisol levels were observed in 12-month-old children with insecure-avoidant and disorganized attachment in response to the SSP (Spangler and Grossmann, 1993). Therefore, we concluded that ICM studies should focus on the identification of protective factors that buffer the negative effects of maternal CM on child stress regulatory function.

### Intergenerational Cycle of Maltreatment and Child Biological Susceptibility – Factors of Genetic Risk and Resilience

Children’s differential susceptibility to their environment must be considered in addition to the effects of parental caregiving quality and other early stressors. The differential susceptibility theory proposes that not all children are affected as much by environmental influences, including parenting (Belsky and van IJzendoorn, 2017). Genetic, stress regulatory, and temperamental features differentiate individuals who are more susceptible from those who are more resilient to the consequences of negative parenting behavior (Bakermans-Kranenburg and van IJzendoorn, 2015). Genetic susceptibility factors likely operate through neurobiological processes (Ellis et al., 2011). Genetic polymorphisms might impact the physiological coupling of mental stress to biological stress response-processes, changing dose-response relationships with effects from cellular up to systemic levels.

Following this perspective, we focused on the role of a common single nucleotide polymorphism (SNP) within the gene coding for the oxytocin receptor (OXTR), namely rs2254298. Previous studies of the rs2254298 genotype showed a gene × environment interaction in the context of intergenerational transmission of risk and resilience (Thompson et al., 2011). Girls carrying at least one A allele were more susceptible to developing symptoms of depression and anxiety if their mothers had a history of recurrent depression. In addition, neuroimaging using functional MRI scans of adolescents demonstrated biological underpinnings that confer increased risk in individuals with at least one A allele in response to early life stress (Marusak et al., 2015). The results of the structural and functional analysis revealed that A allele carriers were characterized by higher bilateral amygdala gray matter volume together with higher amygdala activation during the presentation of emotionally loaded faces (Marusak et al., 2015). Interestingly, there was a positive correlation between the number of stressful life events and amygdala reactivity in A allele carriers only (Toepfer et al., 2017). Variations in brain areas, like the amygdala, affect the activity of the neuroendocrine stress response system (Bourgin et al., 2015; Orem et al., 2019).

We proposed that children carrying the (A) risk allele rs2254298 may be more affected, at least partially, by the negative effects of CM on maternal parenting. Therefore, we investigated the proposed influence of child biological susceptibility in terms of genetic variability at rs2254298 (A vs. G genotype) on child stress regulatory development in the context of ICM.

Another polymorphism in the oxytocin (OXT) gene rs2740210 has been shown to interact with early life adversity. Studies showed that the interaction affected breastfeeding duration (Jonas et al., 2013) and the quality of maternal care independently and in interaction with negative early life experiences (Mileva-Seitz et al., 2013). Previous research in children further revealed that the rs2740210 polymorphism is related to children’s psychological development (Wade et al., 2016; Smarius et al., 2020). In a study with 320 pre-school aged children, a two-marker OXT haplotype, including the rs2740210 SNP, was associated with the amount of externalizing symptoms (Wade et al., 2016). Prospective research showed that children carrying the C (risk) allele were more likely to develop general anxiety symptoms at the age of 5–6 and emotional symptoms at the age of 11–12 when exposed to maternal verbal aggressive behavior in the 13th postnatal week (Smarius et al., 2020).

Based on these findings, we expect that children carrying the C (risk) allele are more likely to demonstrate the negative consequences of maternal CM exposure. Therefore, we investigated the influence of the rs2740210 (C vs. A genotype) on child attachment and disorganized behavior in the context of ICM.

## Aims and Hypotheses

Previous research indicated that the attachment system, maternal social support system, and child biological susceptibility (genetic features and stress reactivity) are central contributors to the path of maladaptation or resilience when mothers have experienced CM. Thus, a deeper understanding of the contribution of each of these factors to ICM is essential to build up prevention models and intervention instruments. In the present study, we aimed to disentangle for the first time in this area of study risk and resilience factors influencing children’s development in the context of maternal CM by using a highly interdisciplinary and prospective study design to test the proposed models.

**FIGURE 1:**
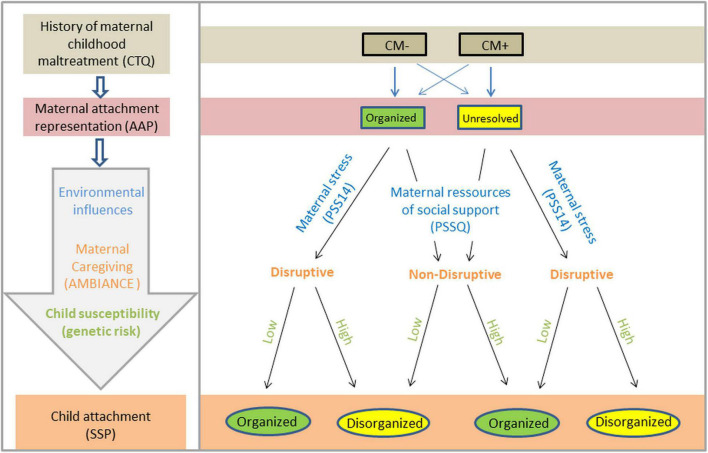
Adapted model from van IJzendoorn and Bakermans-Kranenburg (2019) for the root effects of the intergenerational cycle of maltreatment (ICM) and its effects on the level of attachment in mother-infant dyads. Allelic discrimination of OXTR rs2254298 includes genotypes AA, AG, and GG. At least one A allele is considered a risk genotype for higher stress susceptibility, and a ditochomous analysis (AX vs. GG) was conducted.

For mothers, we explored influencing factors on the relationship between maternal CM and our main study outcomes including maternal attachment representation, maternal disruptive behavior, perceived stress, psychological symptoms, and social support. In children, we explored attachment, autonomic nervous system reactivity, and genetics represented by rs2254298 and rs2740210 – two common polymorphisms in the OXTR and oxytocin peptide gene (OXT), respectively. Then, based on the existing model by van IJzendoorn and Bakermans-Kranenburg (2019; Figure 1) and results from our research consortium, we further established a path model of ICM.

The following hypothesis clusters were tested:

*Hypothesis cluster 1 – Attachment*

H1.1) In comparison to mothers without CM, unresolved attachment is more prevalent in mothers with CM.

H1.2) We expect a significant association between maternal and child attachment, and mothers with unresolved attachment representation are more likely to have children with disorganized than organized attachment.

H1.3) Organized attachment representation is a potential buffer on child attachment in mothers exposed to CM; that is, organized maternal attachment will be associated with child organized attachment.

H1.4) The effect of maternal attachment on child attachment is mediated by maternal caregiving behavior (disruptive/non-disruptive behavior).

H1.5) We expect that higher maternal disruptive behavior is associated with higher child disorganization ratings during the SSP.

H1.6) Higher maternal disruptive behavior is related to a higher increase in HR and a steeper decline in respiratory sinus arrhythmia (RSA) in the child in response to the SSP.

*Hypothesis cluster 2 – Social support*

H2.1) Maternal CM negatively influences the perception of receiving social support from others. In addition, we expect to find a positive association between maternal CM load (CTQ sum score) and maternal psychological problems and distress levels.

H2.2) Social support has an attenuating effect on maternal psychopathology and distress levels. First, we assume that the positive association between maternal CM load (CTQ sum score) and maternal psychological symptoms is mediated by perceived social support.

We also expect that the relationship between maternal CM load (CTQ sum score) and perceived stress in mothers is mediated by perceived social support, such that higher exposure to CM is related to lower social support and higher perceived stress levels.

In addition, we hypothesize that perceived psychological stress also plays a mediating role in the association between maternal CM load and psychopathology.

H2.3) We assume that more perceived social support is associated with lower disruptive behavior.

H2.4) We hypothesize that the use of institutional support by mothers is associated with lower disruptive behavior toward their children.

*Hypothesis cluster 3 – Child genetic features and stress susceptibility*

H3.1) We suggest that carrying at least one A allele (i.e., AA/AG genotype) of the rs2254298 polymorphism in the OXTR gene is associated with a higher stress susceptibility in the offspring as reflected in a higher increase in HR and a steeper decline in RSA during the SSP.

H3.2) We hypothesize that the carrying at least one C allele of the rs2740210 polymorphism (i.e., AC/CC genotype) of the OXTR gene is associated with higher child disorganization ratings assessed during the SSP in dyads where mothers are exposed to CM.

## Materials and Methods

### Study Design and Recruitment of Participants

The project *TRANS-GEN* was funded by the German Federal Ministry of Education and Research BMBF (funding number 01KR1304A) and was conducted between 2013 and 2016. All aspects of the study fulfilled the guidelines of the Declaration of Helsinki. The study protocol was approved by the Local Ethics Board at Ulm University. In total, *N* = 533 women were recruited for study participation within 1 week after parturition at the maternity ward of the Ulm University Hospital (time point *t*_0_). Exclusion criteria for study participation were maternal age under 18 years, severe health problems of the mother or her child, severe complications during parturition, current drug use, history of psychotic disorders, current infections, and insufficient knowledge of the German language at the time of recruitment (Roder et al., 2020; see details, e.g., in Gumpp et al., 2020).

The study employed a prospective, longitudinal design with serial assessments of the mother–child dyads. Follow-up visits were scheduled for 3 months (time point *t*_1_) and 12 months postpartum (time point *t*_2_). After the initial assessment at time point *t*_0_, *n* = 240 mother–child dyads participated at time point *t*_1_ and at time point *t*_2_. The participants in the current report included *n* = 158 mother–child dyads for whom we had complete information on the study variables. The sample demographics, clinical, attachment, and biological characteristics are shown in Table 1.

**TABLE 1:** Demographics and clinical characteristics of mother-child dyads.

Variables	Total *N* = 158
Maternal age at t0 (years), M (SD)	32.61 (4.28)
Sex of the child, n (%)	
*Male*	85 (53.8)
*Female*	73 (46.2)
Highest maternal school education, n (%)	
*No degree*	1 (0.7)
*Secondary school*	7 (4.6)
*Middle school (Realschule)*	32 (20.9)
*Advanced technical college entrance Qualification*	23 (15)
*A-Level*	90 (58.8)
Partnership, n (%)	
*Steady partnership*	152 (99.3)
*No steady partnership*	1 (0.7)
Maternal childhood maltreatment	
*CTQ sum score, M (SD)*	34 (12.15)
*CTQ emotional abuse, M (SD)*	7.23 (3.57)
*CTQ physical abuse, M (SD)*	5.89 (2.78)
*CTQ sexual abuse, M (SD)*	6.13 (3.55)
*CTQ emotional neglect, M (SD)*	8.9 (4.18)
*CTQ physical neglect, M (SD)*	5.84 (1.85)
Maternal attachment (AAP), n (%)	
*Organized*	147 (93)
*Unresolved*	11 (7)
Maternal caregiving (Ambiance), M (SD)	3.68 (1.25)
Maternal perceived stress (PSS14), M (SD)	19.03 (7.78)
Psychological symptoms (BSI), M (SD)	19.08 (17.49)
Social support (PSSQ), M (SD)	136.76 (23.82)
Institutional support^a^, n (%)	
*Yes*	42 (40)
*No*	63 (60)
Child attachment (SSP)^b^, n (%)	
*Organized*	125 (84.5)
*Disorganized*	23 (15.5)
Child Disorganization (D-Score)^c^, M (SD)	3.17 (2.05)
Child stress response HR (% increase)^d^, M (SD)	19.05 (18.32)
Child stress response RSA (% decrease)^e^, M (SD)	−11.02 (33.59)
Child genotype rs2254298^f^	
*GG*	78 (79.59)
*GA/AA*	20 (20.41)
Child genotype rs2740210^f^	
*AA*	49 (50)
*CACC*	49 (50)

*^a^available data from n = 105, ^b^available data from n = 148, ^c^available data from n = 106, ^d^available data from n = 156, ^e^available data from n = 152, ^f^available data from n = 98. M, Mean; SD, Standard deviation; CTQ, Childhood Trauma Questionnaire; AAP, Adult Attachment Projective Picture System; BSI, Brief Symptom Inventory; PSS-14, Perceived Stress Scale-14; PSSQ, Perceived Social Support Questionnaire; SSP, Strange Situation procedure; HR, Heart rate; RSA, Respiratory sinus arrhythmia (RSA) is defined as heart rate variability in synchrony with respiration.*

### Assessment of Maternal Adverse Childhood Experiences

At time point *t*_0_ all women completed the German short version of the *Childhood Trauma Questionnaire* (CTQ, Bader et al., 2009). Here, participants are asked to provide a self-report about their experiences of emotional, physical, or sexual abuse as well as emotional and physical neglect in childhood until the age of 18. The CTQ has five subscales with five items each that are rated on a 5-point Likert scale, higher values indicate a higher CM load (Schury and Kolassa, 2012). The sum score of the 25 item scores (ranging from 25 to 125) was calculated to indicate cumulative maltreatment experiences. Using the cut-off criteria established by Bernstein and Fink (1998) to group mothers as “none,” “low,” “moderate,” or “severe,” the study cohort was split into two groups: mothers who reported none or low, and those who reported moderate or severe CM experiences in at least one CTQ subscale. The latter were categorized as CM+ (*n* = 71); the former were categorized as CM- (*n* = 87). Analyses used the dichotomous variable (CM+/CM-) or the continuous sum CTQ score where appropriate. The results of the prevalence rates of CM and the assessment of CM load classification is summarized in Table 2.

**TABLE 2:** Prevalence rates of childhood maltreatment (CM) and the resulting classification of CM load.

Study cohorts	CM load			

**Total (*N*** = **158); n (%)**	**None**	**Low**	**Moderate**	**Severe**
Emotional abuse	127 (80.4%)	18 (11.4%)	6 (3.8%)	7 (4.4%)
Physical abuse	142 (89.9%)	8 (5.1%)	2 (1.3%)	6 (3.8%)
Sexual abuse	136 (86.1%)	5 (3.2%)	5 (3.2%)	12 (7.6%)
Emotional neglect	102 (64.6%)	35 (22.2%)	13 (8.2%)	8 (5.1%)
Physical neglect	138 (87.3%)	9 (5.7%)	8 (5.1%)	3 (1.9%)

**CM+ only (*n*** = **71)**	**None**	**Low**	**Moderate**	**Severe**

Emotional abuse	40 (56.3%)	18 (25.4)	6 (8.5)	7 (9.9%)
Physical abuse	55 (77.5%)	8 (11.3%)	2 (2.8%)	6 (8.5%)
Sexual abuse	49 (69.0%)	5 (7.0%)	5 (7.0%)	12 (16.9%)
Emotional neglect	15 (21.1%)	35 (49.3%)	13 (18.3%)	8 (11.3%)
Physical neglect	51 (71.8%)	9 (12.7%)	8 (11.3%)	3 (4.2%)

### Assessment of Maternal Attachment Representation

At *t*_1_, all mothers were administered to the *Adult Attachment Projective Picture System* (AAP, George and West, 2012) conducted by trained psychologists. The AAP (George and West, 2001, 2012) is a validated free-response measure that assesses adult attachment representation. It is based on the analysis of narrated “story” responses to a set of seven theoretically derived attachment-related drawings of scenes depicting solitude, illness, separation, death, and potential maltreatment. Individuals are asked to tell a story for each picture following a standardized set of probes. All AAPs were transcribed verbatim from audio recordings for analysis and attachment classification.

Each picture stimulus is coded for content and defensive processes (for a more detailed description of the coding and classification procedure see George and West, 2011, 2012). The AAP classifies the four established attachment categories: secure, insecure-dismissing, insecure-preoccupied, and unresolved attachment. For our present study, attachment representations of the mothers were divided into two major classifications “organized” and “unresolved.” Organized attachment includes secure, dismissing, and preoccupied classifications; here, any frightening or threatening material (e.g., desperately alone, death, attack, abuse, helplessness, danger, failed protection, isolation) that may appear in the story is contained (i.e., resolved). The unresolved attachment refers to a group of individuals who are not able to regulate and contain or reorganize stories that evidence frightening or threatening material. In short, unresolved individuals are flooded and dysregulated by their attachment fears.

Studies demonstrate good psychometric properties of the AAP in adults (Buchheim et al., 2008; Benoit et al., 2010; George and West, 2012) and adolescents (Gander et al., 2017) by showing high inter-rater reliability (George and West, 2001; Buchheim et al., 2008; Benoit et al., 2010), discriminant validity in controls and clinical patients and test–retest reliability (George and West, 2001, 2012). Also, a high concurrent validity with the *Adult Attachment Interview* (AAI; George et al., 1985; Main and Goldwyn, 1998) was demonstrated in several independent samples (George and West, 2012; Buchheim et al., 2018; Gander et al., 2022).

Maternal AAP classification was performed by two independent certified judges. Inter-rater reliability showed significant concordance for the four-group classification (κ = 0.95, 95%-confidence interval [0.88, 1.04], *p* < 0.001), and for the two-group classification (organized vs. unresolved, κ = 0.96), 95%-confidence interval [0.91, 1.00], *p* < 0.001.

### Assessment of Child Attachment

Child attachment and maternal behavior were assessed at 12 months (*t*_2_) in a laboratory setting using the *Strange Situation Procedure* (Ainsworth, 1978). The SSP is a standardized procedure to observe mother–child interaction in an attachment context. The procedure involves a series of eight episodes lasting approximately 3 min each, whereby the child is increasingly stressed by the introduction of a stranger, and two separations from and reunions with the mother. Children are classified as secure, insecure (avoidant, ambivalent), or disorganized (highly insecure). The disorganized behavior (D-score) is rated along a scale focusing on, e.g., the degree of sequential and simultaneous display of contradictory behavior patterns; incomplete, and interrupted movements and expressions; stereotypies, freezing, stilling, and slowed movements and expressions, and direct indices of disorganization and disorientation. The current study used two groupings: organized (secure, avoidant, ambivalent) and disorganized. Moreover, children rated at or above the D-score 5 (midpoint of the Likert scale ranging from 1 to 9) were placed in the disorganized group (Main and Solomon, 1986). This study also used the D-score for analysis.

The good psychometric properties of the SSP like high inter-rater-agreement are well-documented (e.g., De Wolff and van IJzendoorn, 1997; Solomon and George, 1999). The SSP was videotaped for later analysis. All videos were classified and rated by Judith Solomon, one of the co-developers of the category of the disorganized attachment pattern (Main and Solomon, 1986).

### Assessment of the Child’s Autonomic Nervous System Response

The dyads participated in the laboratory procedure between 10 a.m. and 1 p.m. Upon arrival, the experimenter explained the procedures to the mothers, while the dyad was in a “resting and relaxing phase,” which lasted approximately 15–20 min. During this time, mothers were asked to connect the wireless lightweight mobile unit with seven disposable spot electrodes onto their child’s chest skin. Before starting the SSP, the dyads listened together to a digitally recorded lullaby to calm down.

During the SSP, child HR, and RSA were determined continuously using a wireless lightweight mobile unit (Mindware Technologies, Gahanna, United States). RSA is the naturally occurring variation in HR that occurs during the breathing cycle. RSA is directly proportional to HRV and can be used to assess cardiac parasympathetic innervation, while HR is associated with both sympathetic and parasympathetic cardiac innervation. Higher stress levels are normally related to an increase in HR (due to parasympathetic withdrawal and higher sympathetic nervous system activity) and a decrease in RSA (due to parasympathetic withdrawal; Beauchaine, 2015; Kim et al., 2018). Before statistical analysis, the data were filtered and scored using the mindware software (BioLab 3.1 1.0J; Mindware Technologies, United States). Signal artifacts due to body movements, vocalizations, or close physical contacts were excluded from the datasets. Each segment of the dataset was checked and corrected for inaccurate R-peak detections by trained coders following previous reports (Roder et al., 2020). Each of the eight episodes of the SSP was divided into 30-s segments, the first six of which were used for statistical analysis. If there were fewer than six segments available, data from the total recording was used. The data-cleaning procedures, including surveillance at random, were adapted to previously described procedures (Roder et al., 2020). Measures of reactivity were determined by calculating the percent change in HR and RSA between baseline (episode 1) and the episode where the stranger comes into the room following the second separation instead of the mother (episode 7). Prior analysis from our research consortium indicated that episode 7 showed the highest autonomic nervous system stress reactivity in children (Roder et al., 2020).

### Assessment of Maternal Caregiving (Disruptive/Non-disruptive Behavior)

The quality of maternal caregiving behavior was analyzed using the *Atypical Maternal Behavior Instrument for Assessment and Classification* (*AMBIANCE*; Bronfman et al., 1992) and performed in the context of the SSP at timepoint *t*_2_. Lyons-Ruth et al. (1999) developed the *AMBIANCE* scale to elaborate the work by Main and Hesse (1990) to assess mothers’ anomalous, disruptive interactive behavior with their children and is a widely used and well-validated measure with good psychometric properties. The scale evaluates disrupted maternal behaviors on five dimensions: (1) affective communication errors, (2) role/boundary confusion, (3) disorganized/disoriented behaviors, (4) negative/intrusive behavior, and (5) withdrawal. Behaviors on each of the dimensions are rated on a 7-point *Likert* scale and a sum score of the level of disruption is determined. The level of disrupted communication was assigned based on the frequency and intensity of all disrupted behaviors mothers displayed in the course of the interaction with their children. The current study used the *AMBIANCE* sum score of disruption. A rater trained to reliability by the *AMBIANCE* developers scored all interactions and was blind to all other data collected in the study.

### Maternal Perceived Stress

Perceived psychological stress over the past month was assessed using the *Perceived Stress Scale* (PSS-14, Cohen, 1988) at *t*_1_ and *t*_2_. The PSS-14 is the most widely used psychological self-report instrument for measuring perceived stress. It assesses the degree to which life situations are appraised as stressful, tapping how unpredictable, uncontrollable, and overloaded individuals find their lives over the last month using 14-items rated on a 5-point *Likert* scale. We observed a moderate and highly significant (*r* = 0.55, *p* < 0.001) correlation of the PSS-14 score between *t*_1_ and *t*_2_; therefore, the mean value of the two time points was used for the statistical analysis.

### Maternal Psychological Symptoms

The *Brief Symptom Inventory* (BSI, Wideman et al., 2013) was assessed at *t*_1_ and is a self-report instrument including 53 items measuring psychological symptoms during the last 7 days. It covers the nine primary symptom dimensions: somatization, obsessive-compulsive, interpersonal sensitivity, depression, anxiety, hostility, phobic anxiety, paranoid ideation, and psychoticism. Items are answered on a 5-point scale ranging from 0 (not at all) to 4 (extremely). The BSI sum score was used for analysis.

### Social Support (Postpartum Social Support Questionnaire and Institutional Support)

Social support was assessed at *t*_1_ and *t*_2_ using the *Postpartum Social Support Questionnaire* (PSSQ, Hopkins and Campbell, 2008). The PSSQ is a 50-item self-report that measures social support received by women in recent motherhood; it evaluates emotional and instrumental social support received from the spouse-partner, parents, in-laws, and other family and friends. The PSSQ scores at *t*_1_ and *t*_2_ were significantly correlated (*r* = 0.79, *p <* 0.001), so the mean value of the two time points was used in our statistics. In addition to the PSSQ, the use of parental-counseling services (institutional support) was assessed by an interview and coded as a dichotomous variable (yes/no).

### Genotyping

The child’s umbilical cord blood was used for the isolation of DNA. The quantity and purity of isolated DNA were assessed with the high-sensitivity DNA quantification kit and a Qubit fluorometer (ThermoFisher Scientific, United States). Genotyping of rs2254298 and rs27401210 was conducted using a Taqman assay in combination with a QuantStudio 6 qPCR device following the protocol of the manufacturer (ThermoFisher Scientific, United States). Allelic discrimination of samples for the rs2254298 (GG vs. GA/AA) and rs2740210 SNP (AA vs. CA/CC) was performed with the QuantStudio 6 genotyper operating software (ThermoFisher Scientific, United States). The results of the genotyping were translated into dichotomous variables (GG vs. GA/AA and AA vs. CA/CC, respectively) for statistical analysis.

**FIGURE 2:**
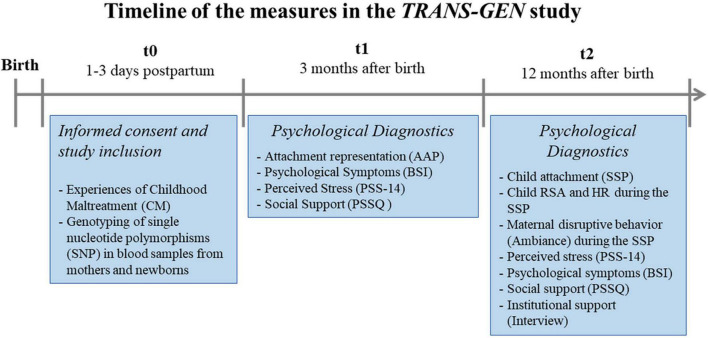
Time line of the measures in the *TRANS-GEN* Study.

Timeline of the measures used and assessed in the *TRANS-GEN* study are summarized in Figure 2.

### Processing of Raw Data and Statistical Analyses

Database generation and data analysis were conducted using the SPSS statistical software (SPSS 26.0, Inc., Chicago, IL, United States). Pearson’s correlations or Spearman’s rank correlation were used for bivariate analyses depending on the scaling type of the raw data. Chi-square tests of independence were performed to examine the association between the dichotomous variables, maternal exposure to CM (CM+/CM-) and maternal attachment representation (organized, unresolved), and the association between maternal and child attachment (organized, disorganized). The PROCESS (Hayes, 2008) macro model 4 was used to test for the mediating role of maternal disruptive behavior on the relationship between maternal and child attachment. Macro model 6 was applied to assess the potential mediating roles of social support (PSSQ) and perceived stress (PSS14) on the relationship between the CTQ sum score and psychological symptoms (BSI sum score). We conducted ordinary least squares path analyses using 10,000 bootstrapping samples and a 95% confidence interval (CI). Univariate analysis were applied to test for the effect of the rs2254298 SNP on the child’s autonomic nervous system response (HR and RSA reactivity percentage between e1 and e7 of the SSP) and the effect of the rs2740210 SNP on child disorganized behavior (D-score). Here, the interaction terms rs2254298 × CM+/CM- and rs2740210 × CM+/CM- were included to test for effect modification by maternal CM exposure. Chi-square tests of independence were performed to test the effect of the rs2740210 SNP on child attachment (organized/disorganized) in the total cohort and in the subsample of mothers with CM (CM+). For the analytical aspects of the path analysis, the software Mplus (Muthén and Muthén, 1998) was used. We decided to perform a step-wise approach in the analyses of the individual parameters assessed in the given study. Then we conducted maximal likelihood path analyses and used the *χ^2^* test, comparative fit index (CFI) and the root mean square error of approximation (RMSEA) to decide on model fit. The significance level was set at *p* < 0.05 for all analyses.

## Results

### Correlation Analysis

Intercorrelations of all study variables are shown in Supplementary Table 1 for the total cohort and in Supplementary Table 2 for the subsample of mothers with CM only (CM+). In the following sections, we present the results of hypotheses testing.

### Hypotheses Testing

#### Attachment

Our first set of hypotheses examined the attachment system. We first examined the prevalence of unresolved attachment in CM mothers, then tested the correspondence of maternal and child attachment by focusing on the impact of maternal organized attachment on the child’s attachment as a potential resilience factor. We then explored if the effect of maternal attachment on child attachment is mediated by maternal caregiving behavior (disruptive/non-disruptive behavior) and how maternal disruptive behavior affects the child’s disorganized behavior and stress reactivity.

##### Unresolved Attachment Is More Prevalent in Mothers With Childhood Maltreatment Compared to Mothers Without Childhood Maltreatment

Hypothesis H1.1. was examined using a *χ^2^* test of independence testing the association between maternal exposure to CM (CM+/CM-) and maternal attachment representation (organized, unresolved). The results showed that from the mothers exposed to CM (*n* = 71), *n* = 11 (15.5%) showed unresolved attachment, while none of the non-exposed mothers (*n* = 87) showed unresolved attachment (*n* = 0, 0%), indicating a significant higher percentage of mothers with unresolved attachment in the CM+ group, *x*^2^ (1, *N* = 158) = 14.49, *p* < 0.001.

##### Correspondence of Maternal and Child Attachment

Hypothesis H1.2 examined the distribution of children with disorganized attachment in the sample. The results showed that from mothers with organized attachment (*n* = 139), only *n* = 18 children (13%) showed disorganized attachment. The prevalence of children with disorganized attachment (*n* = 5, 50%) was much higher in the group of mothers with unresolved attachment (*n* = 10). The statistical between-group comparison revealed a significant effect of this distribution, indicating a higher percentage of children with disorganized attachment in the group of unresolved mothers than organized mothers, *x*^2^ (1, *N* = 148) = 9.70, *p* = 0.002.

##### Organized Maternal Attachment in Mothers With Childhood Maltreatment Is a Potential Buffer on Child Attachment

As hypothesized in H1.3, we could demonstrate in a subgroup analysis considering only mothers with CM (CM+) that organized maternal attachment representation (AAP) was associated with organized child attachment patterns (SSP) *x*^2^ (1, *n* = 66) = 5.84, *p* = 0.02. In the group of mothers with an organized attachment (*n* = 56), *n* = 47 children (84%) showed an organized attachment, while *n* = 9 children (16%) were disorganized. In contrast, the group of mothers with an unresolved attachment (*n* = 10) included *n* = 5 children with a disorganized attachment (equals 50%) and *n* = 5 with an organized attachment.

##### The Effect of Maternal Attachment on Child Attachment Is Mediated by Maternal Caregiving Behavior (Disruptive/Non-disruptive Behavior)

Next, we investigated the mediating role of maternal disruptive behavior on the relationship between maternal attachment representation and child attachment. Mediation analysis for testing H1.4 showed a direct effect of maternal attachment representation on child attachment (*b* = 1.76, CI 95%: 0.42–3.11, *p* = 0.01), but no indirect, or mediating effect, *via* maternal disruptive behavior (*b* = 0.19, CI 95%: −0.12–0.62, *p* > 0.05). These results indicate that, in contrast to maternal attachment representation, maternal disruptive behavior did not sufficiently contribute to explaining child attachment.

##### The Impact of Maternal Disruptive Behavior on Disorganized Behavior of the Child

As hypothesized in H1.5, we confirmed a significant correlation between maternal AMBIANCE scores and D-scores in the SSP of the children (*r* = 0.26, *p* = 0.008) demonstrating that higher maternal disruptive behavior toward their child was associated with a higher amount of disorganized behavior in their child.

##### The Influence of Maternal Disruptive Behavior on Child Stress Reactivity

For hypothesis H1.6, we investigated the relationship between maternal disruptive behavior and the stress reactivity of the child using HR and RSA. We found a significant correlation between AMBIANCE and HR (*r* = 0.17, *p* = 0.04) and AMBIANCE and RSA (*r* = −0.17, *p* = 0.04). The correlation remained significant in the CM+ subgroup for RSA (*r* = −0.27, *p* = 0.03), but there was no longer a significant association between AMBIANCE and HR (*r* = 0.13, *p* = 0.30).

#### The Social Support System

Our second set of hypotheses examined the maternal support system. We first investigated the impact of CM load (CTQ sum score) on perceived maternal stress, psychological problems, and perceived social support. We then examined the influence of the social support system in the context of ICM. Two types of social support were explored: (1) social support from the partner, friends, and family assessed by the PSSQ and (2) the use of institutional support by the mothers.

##### Childhood Maltreatment and Its Impact on Perceived Maternal Stress, Psychological Problems, and Perceived Social Support

As hypothesized in H2.1, we found positive associations between CM load (CTQ sum score) and maternal perceived stress (*r* = 0.31, *p* < 0.001) and psychological symptoms (*r* = 0.48, *p* < 0.001). We also observed a positive association between perceived stress levels and psychological symptoms (BSI sum score) (*r* = 0.73, *p* < 0.001) and confirmed a negative association between maternal CM load and perceived social support, *r* = −0.42, *p* < 0.001.

##### Social Support and Perceived Stress and Their Mediating Role in the Association Between Childhood Maltreatment and Psychological Symptoms

As expected in H2.2, greater social support was significantly associated with lower perceived stress, *r* = −0.31, *p* < 0.001 and less psychological symptoms, *r* = −0.38, *p* < 0.001. In the subgroup of CM+ mothers, the association between social support and psychological symptoms (*r* = −0.24, *p* = 0.047) was still significant, but the relationship between social support and perceived stress was no longer significant (*r* = −0.17, *p* = 0.16).

To further investigate these associations, we tested the possibility that the relationship between maternal CM load (CTQ sum score) and maternal psychopathology was mediated by social support and perceived stress. Mediation analysis revealed that both the direct effect of CM load on psychological symptoms (*b* = 0.37, CI 95%: 0.20–0.53, *p* < 0.001) and the indirect effect *via* social support and perceived stress was significant (*b* = 0.08, CI 95%: 0.03–0.15, *p* < 0.05), indicating a partial mediating effect of social support and perceived stress on the development of psychological symptoms. In this serial mediation, CM load was associated with lower social support (*b* = −0.81, CI 95%: −1.1 to −0.53, *p* < 0.001) and lower social support was related to more perceived stress (*b* = −0.07, CI 95%: −0.13 to −0.02, *p* = 0.007). In addition, higher perceived stress levels were associated with more psychological symptoms (*b* = 1.40, CI 95%: 1.15–1.64, *p* < 0.001).

##### The Influence of Social Support on Maternal Caregiving (Disruptive Behavior)

Following the model by van IJzendoorn and Bakermans-Kranenburg (2019) (see the adapted model in Figure 1), we examined the influence of social support on maternal caregiving (disruptive behavior) using correlation analyses. We observed no significant effect of social support on maternal disruptive behavior in both the total cohort (*r* = −0.13, *p* = 0.12) and the CM+ subgroup (*r* = −0.19, *p* = 0.11).

##### Effects of Institutional Support

We hypothesized in H2.4 that maternal use of institutional support would be associated with lower disruptive behavior towards their children. Our results demonstrate a significant association between the reported use of parental-counseling services (yes/no) and higher disruptive behavior (*r* = 0.20, *p* = 0.04), indicating that these mothers sought institutional support more frequently.

#### Child Genetic Susceptibility Related to Oxytocin Receptor rs*2254298* and Oxytocin rs*2740210*

Our third set of hypotheses examined genetic risk factors within the OXTR gene of the child in a two-step approach. First, we investigated the SNP rs2254298 on child autonomic nervous system response during the SSP, followed by the influence of rs2740210 on child attachment development.

##### The Influence of rs*2254298* on Child Autonomic Nervous System Response During the Strange Situation Procedure

Initially, we tested the effect of rs2254298 on the child’s autonomic nervous system reactivity. Univariate analysis indicated there was no effect of carrying the A (risk) allele on RSA [*F*(1, 91) = 1.55, *p* = 0.22]; *M* (*SD*) A (risk) allele carriers = −18.62% (38.88), *M* (*SD*) non-risk allele carriers = −7.92% (32.68) and HR reactivity [*F*(1, 95) = 0.001, *p* = 0.97]; *M* (*SD*) A (risk) allele carriers = 19.56% (19.96), *M* (*SD*) non-risk allele carriers = 19.40% (18.51) during the SSP. However, when we tested for effect modification by maternal CM (CM+/CM-), we observed a significant interaction between maternal CM exposure and the rs2254298 SNP on the HR response [*F*(1, 93) = 6.04, *p* = 0.016; *M* (*SD*) CM+/A (risk) allele carriers = 27.95% (15.08); *M* (*SD*) CM-/A (risk) allele carriers = 13.97% (21.40); *M* (*SD*) CM+/ non-risk allele carriers = 14.63% (15.47); CM-/ non-risk allele carriers = 23.59% (20.07)] and the RSA response [*F*(1, 89) = 7.64, *p* = 0.007; *M* (*SD*) CM+/A (risk) allele carriers = -37.16% (27.68); *M* (*SD*) CM-/A (risk) allele carriers = −6.26% (41.30); *M* (*SD*) CM+/ non-risk allele carriers = 0.52% (28.92); CM-/ non-risk allele carriers = −15.28% (34.32)]. This result indicates that for children of CM+ mothers, the autonomic nervous system stress response was moderated by the rs2254298 polymorphism (i.e., a higher HR increase and a steeper decline in RSA was observed in children with CM+ mothers that carried the risk allele).

##### The Influence of rs*2740210* on Child Attachment Development

We also tested the effect of the rs2740210 polymorphism on child attachment and disorganized behavior. Results for the total cohort showed a trend toward a significant effect of carrying the C (risk) allele on child attachment, *x*^2^ (1, *N* = 93) = 3.03, *p* = 0.08. In the group of children carrying the C (risk) allele (*n* = 45), *n* = 12 children (27%) showed unresolved attachment, while *n* = 33 children (73%) displayed organized attachment. In contrast, in the group of non-risk allele carriers (*n* = 48), *n* = 6 children (12.5%) showed unresolved attachment and *n* = 42 an organized attachment (87.5%). In the subgroup of children with mothers that experienced CM (CM+ group) there was no significant effect of carrying the C (risk) allele on child attachment, *x*^2^ (1, *N* = 41) = 2.45, *p* = 0.12. In C (risk) allele carriers (*n* = 16), *n* = 6 children (37.5%) showed unresolved attachment, while *n* = 10 children (62.5%) displayed organized attachment. In the group of non-risk allele carriers (*n* = 25), *n* = 4 children (16%) showed unresolved attachment and *n* = 21 an organized attachment (84%).

We observed a significant effect of the C (risk) allele on child D-score [*F*(1, 68) = 6.8, *p* = 0.01], indicating that children carrying the risk allele showed more disorganized behavior (*M* (*SD*) C (risk) allele carriers = 3.87 (2.20); *M* (*SD*) non-risk allele carriers = 2.67 (1.63)] during the SSP. When we tested for effect modification by maternal CM+/ CM-, we observed no significant interaction between maternal CM+/CM- and the rs2740210 SNP on child attachment [*x*^2^ (1, *N* = 93) = 0.015, *p* = 0.90] and D-score [*F*(1, 66) = 0.002, *p* = 0.97; *M* (*SD*) CM+/C (risk) allele carriers = 4.14 (2.40); *M* (*SD*) CM-/C (risk) allele carriers = 3.67 (2.10); *M* (*SD*) CM+/ non-risk allele carriers = 2.87 (1.74); CM-/ non-risk allele carriers = 2.44 (1.22)].

### Path Analysis of the Intergenerational Cycle of Maltreatment Model

Using the (correlational) results presented in the Supplementary section, we developed a model of ICM by adapting the model proposed by van IJzendoorn and Bakermans-Kranenburg (2019). Child outcome variables were child attachment (organized/disorganized) and child autonomic nervous system response (change in RSA). Other variables that were included in the model were maternal CM (CTQ sum score), maternal attachment representation (organized/disorganized), maternal caregiving (level of disruptive behavior), perceived stress, psychological symptoms, social support, and child genotypes rs2254298 and rs2740210. The model showed a good fit to the data with good fit indices [*x*^2^ (28) = 25.50, *p* = 0.65, CFI = 1.00, RMSEA = 0.00, *p* = 0.88]. The path analysis was repeated for the CM+ sub-cohort and also here the model showed good fit indices [*x*^2^ (28) = 25.50, *p* = 0.26, CFI = 0.94, RMSEA = 0.06, *p* = 0.41].

**FIGURE 3:**
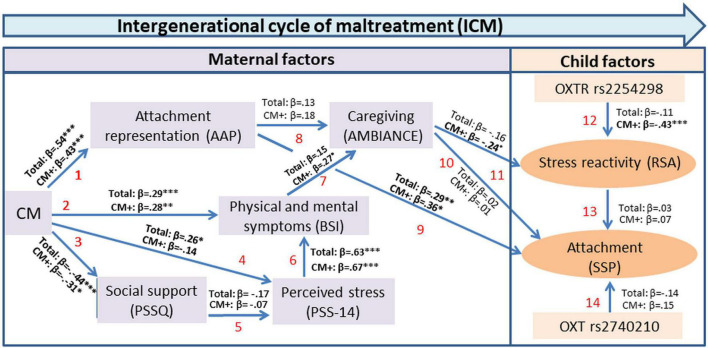
Extended empirical pathway ICM model based on the theoretical model for the intergenerational transmission of attachment by van IJzendoorn and Bakermans-Kranenburg (2019) shown in Figure 1.

Next, we report the results of the individual paths of the model (see also Figure 3).

1. (*CM to AAP*): CM (CTQ) showed a positive and statistically significant association with the attachment representation (AAP), indicating that higher maltreatment was associated with a higher likelihood for unresolved attachment (β = 0.54, *p* < 0.001). The same findings resulted from the CM+ subgroup analyses (β = 0.43, *p* < 0.001).

2. (*CM to BSI*): The analysis of the association between CM and psychological symptoms (BSI) revealed a significant effect (β = 0.29, *p* < 0.001), which highlights a dose-dependent association between adverse life stress and psychological symptoms. This finding was replicated in the CM+ subgroup (β = 0.28, *p* = 0.005).

3. (*CM to PSSQ*): A significant and negative association was found between maternal CM and the self-reported resources of maternal social support (β = −0.44, *p* < 0.001). The finding was comparable to the results for the CM+ subgroup (β = −0.31, *p* = 0.03).

4. (*CM to PSS14*): In the total cohort, a significant and positive association was found between maternal CM and self-reported perceived maternal stress (β = 0.26, *p* = 0.01), but no significant association in the CM+ subgroup (β = 0.14, *p* = 0.37).

5. (*PSSQ to PSS14*): The association between social support and self-reported perceived maternal stress was not statistically significant (β = −0.17, *p* = 0.11). The finding was similar for the CM+ subgroup (β = −0.07, *p* = 0.64).

6. (*PSS14 to BSI*): Self-reported perceived maternal stress showed a positive and statistically significant association with psychological symptoms (β = 0.63, *p* < 0.001). The finding was the same for the CM+ subgroup (β = 0.67, *p* < 0.001).

7. (*BSI to AMBIANCE*:) Psychological symptoms were not significantly related to maternal disruptive behavior (β = 0.15, *p* = 0.14). However, this association showed a trend toward significance for the CM+ subgroup (β = 0.27, *p* = 0.06).

8. (*AAP to AMBIANCE*): Maternal attachment representation was not significantly associated with maternal disruptive behavior both in the total cohort (β = 0.13, *p* = 0.21) and the CM+ subgroup (β = 0.18, *p* = 0.21).

9. (*AAP to SSP*): Maternal attachment representation (organized/unresolved; AAP) showed a positive and statistically significant association with the child attachment pattern (organized/disorganized; SSP; β = 0.29, *p* = 0.003). The finding was similar for the CM+ subgroup (β = 0.36, *p* = 0.01).

10. (*AMBIANCE to SSP*): Maternal disruptive behavior was not significantly associated with child attachment patterns (SSP) in neither the total cohort (β = 0.02, *p* = 0.86) nor the CM+ subgroup (β = 0.01, *p* = 0.95).

11. (*AMBIANCE to RSA*): Maternal disruptive behavior did not show a significant association with stress reactivity in children (change in RSA) in the total cohort (β = −0.16, *p* = 0.14). However, this association showed a trend toward significance in the CM+ subgroup (β = −0.24, *p* = 0.07).

12. (*OXTR* rs2254298 *genotype on RSA*): Child rs2254298 OXTR genotype was not significantly associated with stress reactivity in children (change in RSA; β = 0.11, *p* = 0.26). However, the association was significant in the CM+ subgroup (β = 0.43, *p* = 0.001).

13. (*RSA on SSP*): Children’s stress reactivity (change in RSA) was not significantly associated with child attachment (SSP) in neither the total cohort (β = 0.03, *p* = 0.76) nor the CM+ subgroup (β = 0.07, *p* = 0.67).

14. *(OXTR rs2740210 genotype on SSP)*: Both in the total cohort (β = 0.14, *p* = 0.16) and CM+ subgroup (β = 0.15, *p* = 0.38) there was no significant association between the rs2740210 OXTR genotype and child attachment.

## Discussion

### Study Approach and Aims of the Present Study

The present study tested and replicated a proposed model of the intergenerational transmission of attachment (van IJzendoorn and Bakermans-Kranenburg, 2019) and extended it by studying maternal CM experiences using data collected from a cohort of 158 mother–child dyads. This innovative approach combined examining the role of the attachment system, social support system, and child genetic susceptibility. The prospective design provided a more comprehensive understanding of the intergenerational transmission of CM than previous studies and allowed us to determine potential protective factors that could help to disrupt intergenerational effects.

### Discussion of the Study Results

We now discuss our findings in relation to our hypotheses and their contribution to the literature.

#### Attachment System

##### A History of Childhood Maltreatment Is Associated With a Higher Probability of Unresolved Maternal Attachment

As expected, we found a strong significant association between CM experiences and unresolved attachment in mothers. This result is consistent with the results of previous studies using different samples representing various degrees and types of reported maltreatment (Bakermans-Kranenburg and van IJzendoorn, 2009; Buchheim and Diamond, 2018; Gander et al., 2018, 2020, 2021). The prevalence rate in our study cohort with *N* = 158 participants was 44.9% CM, determined using the recommended cut-off classifications (Koenig et al., 2016). The most prevalent type of CM was emotional neglect (35.44%). This rate is comparable to the prevalence rates of emotional neglect reported in other German community samples (Witt et al., 2017). One of the most prevalent and most rapidly increasing forms of early life maltreatment is emotional neglect, thought to shape social dysfunction in adults (Müller et al., 2019). Subic-Wrana et al. (2011) demonstrated that psychosomatic patients with experiences of emotional neglect showed a diminished ability to contain or integrate evidence of trauma in their AAP narratives. This pattern was replicated in our sample for mothers exposed to CM.

##### Correspondence of Maternal and Child Attachment

We found that maternal attachment representation was significantly associated with child attachment. There was a significant correspondence between the mothers’ organized and unresolved categories and analogous children’s attachment groups. These findings suggest that maternal unresolved attachment is a potential risk factor for child attachment disorganization. We note, however, that the proportion of disorganized children in our study was relatively low, thus comparable to non-clinical samples rather than clinical risk samples (van IJzendoorn et al., 1999).

##### Organized Maternal Attachment in Mothers With Childhood Maltreatment as a Resilience Factor

The study explored the effects of maternal resilience. The subgroup analysis for CM+ mothers demonstrated a robust association between organized attachment for mothers and children. We suggest that maternal organized attachment despite having experienced CM acts as a protective factor for child attachment.

Organized attachment in children is characterized by the ability to establish proximity to the caregiver during a stressful attachment situation; the different strategies observed in the SSP for secure, avoidant, and ambivalent children are considered to be at least minimally regulating and keep attachment behavior and the relationship organized (Main, 1990). In contrast, disorganized attachment is characterized by contradictory and unintegrated responses when comfort is needed, for example, freezing, huddling on the floor when under stress (Main and Solomon, 1986; Lyons-Ruth and Jacobvitz, 2016). In adults, organized attachment at the representational level is characterized by the ability to regulate and contain activated attachment fears and threats. Unresolved adults remain overwhelmed and representational defences break down (George and West, 2012). Most attachment-based intervention programs aim to enhance parents’ regulation processes by bolstering reflection to improve sensitivity and caregiving behavior to interrupt the transmission of insecure and unresolved attachment to the next generation (Steele and Steele, 2018). Therefore, the improvement from unresolved to organized attachment representations shown in previous clinical studies (Buchheim et al., 2017; Buchheim and Diamond, 2018) should be one intervention goal for individuals with a history of CM.

Important to note, this finding replicates studies on the intergenerational transmission of attachment (Fonagy et al., 1991; Benoit and Parker, 1994; van IJzendoorn, 1995; Steele et al., 1996; Behrens et al., 2016; van IJzendoorn and Bakermans-Kranenburg, 2019). This is the first study to demonstrate analogous patterns using the AAP in a large cohort of mother–child dyads as compared with the more widely used *AAI* (Main and Goldwyn, 1998). Convergent validity with the SSP adds to studies demonstrating the good psychometric properties of the AAP (George and West, 2012).

##### The Mediating Role of Caregiving in the Intergenerational Transmission of Attachment

The mediation analysis revealed a direct effect of maternal attachment representation on child attachment; however, maternal caregiving (disruptive behavior) was not a mediator for this effect neither for the total sample nor the CM+ subgroup. This finding is comparable with other studies on the transmission gap (Verhage et al., 2016; van IJzendoorn and Bakermans-Kranenburg, 2019), though using the AMBIANCE Scale (Bronfman et al., 1992) and not the Ainsworth scale (Ainsworth, 1978) for assessing maternal caregiving quality and its mediating role. The rationale for this was, that meta-analytic work found maternal insensitivity to be an insufficient antecedent of disorganized attachment, with insensitivity explaining only a small proportion of variance in this outcome (van IJzendoorn et al., 1999; Verhage et al., 2016). In contrast and adequate for our approach the AMBIANCE scale does capture anomalous behaviors exhibited by caregivers in disorganized attachment relationships (Lyons-Ruth et al., 1999).

Moreover, we agree with the view by van IJzendoorn and Bakermans-Kranenburg (2019) that further studies should include other mediating factors as plausible candidates to explain the gap beyond parental caregiving like sensitivity such as support, protective parenting, synchrony, and examining the repair of mismatches in parent–child interactions.

##### The Impact of Maternal Disruptive Behavior on Disorganized Behavior of the Child

As hypothesized, we confirmed a direct significant relationship between maternal disruptive and mismatched behavior toward the child (e.g., affective communication errors, role or boundary confusion, disoriented behaviors, intrusive behavior, or withdrawal) and children’s disorganized behavior (i.e., freezing, huddling on the floor, and disoriented behaviors). This finding is in line with meta-analysis results reported by Madigan et al. (2006), which confirmed a significant relationship between maternal disrupted communication and child disorganized attachment. Given the association between disorganized attachment and later mental health outcomes, interventions should target enhancing parental sensitivity, reducing disorganizing, disruptive interactions between caregivers and their children, promoting practical support (Wright and Edginton, 2016), and education about child development (Steele and Steele, 2018).

#### Social Support System

##### Childhood Maltreatment and Its Impact on Perceived Maternal Stress, Psychological Problems, and Perceived Social Support

As predicted, mothers reporting CM reported receiving lower levels of social support. This finding is consistent with previous observations from our pilot study (Schury et al., 2017b) as well as other studies (Colman and Widom, 2004). Sperry and Widom (2013) proposed that these individuals’ perceptions of poor availability of social support reflect long-standing biases in their social expectations because of their histories of lack of supportive caregiving. However, we have to state, that our sample did not contain mothers with a severe to extreme CM load. We agree with others (Sperry and Widom, 2013) that efforts to better understand the role of support that may enhance mothers’ abilities to develop and maintain supportive friendships should be considered when developing preventive child maltreatment interventions.

##### Social Support and Perceived Stress and Their Mediating Role

We expected and confirmed that perceived social support can be an important factor for attenuating maternal problems. Greater perceived social support was significantly associated with lower reported maternal stress and fewer psychological symptoms. Moreover, our mediation analysis demonstrated that both social support and perceived stress could mediate the effect of CM exposure on the development of psychological symptoms. This finding is consistent with other studies (Schumm et al., 2006; Afifi and MacMillan, 2011; Salazar et al., 2011; Evans et al., 2013), highlighting the need to assess both social support and perceived stress to fully describe their resilience synergy in attenuating the effects of ICM.

##### The Influence of Social Support on Maternal Caregiving

We did not observe a significant effect of social support on maternal caregiving behavior (disruptive behavior). This finding is in contrast to previous studies suggesting that mothers perceiving themselves as being well supported showed higher sensitivty toward their children (Kivijarvi et al., 2004; Shin et al., 2006; Kim and Kim, 2009) and were more likely to experience personal competence, better coping behaviors, and high levels of self-esteem (Emmanuel et al., 2011). An explanation for this discrepancy could be the application of different instruments for the assessments of social support and maternal caregiving quality.

##### Effects of Institutional Support

We also examined the effects of institutional support, a factor suggested in the model by van IJzendoorn and Bakermans-Kranenburg (2019) on maternal caregiving. However, the direction of the observed association was unexpected. Mothers with higher disruptive scores reported more requests for institutional support. Disruptive mothers, especially mothers of children with disorganized attachment, are often distressed and, therefore, may have a greater need for parenting support that external support structures typically provide. However, we cannot rule out that our results were different if we would have measured maternal caregiving quality more often covering different mother-child interactions and measures (including, e.g., the sensitivity scale by Ainsworth, 1978) in the course of our longitudinal study.

#### Child Biological Susceptibility and Stress Reactivity

##### The Influence of rs*2254298* on Child Stress Responsivity

We showed for the first time in the literature an effect of a gene × environment interaction between maternal CM and child rs2254298 polymorphism on child HR and the RSA response during the SSP. There was a more pronounced cardiac stress response in children from CM exposed mothers that carried the risk (A) allele. This finding confirmed our hypothesis and is in line with previous reports demonstrating the role of the rs2254298 polymorphism on the development of brain structures important for regulating the neuroendocrine stress response (Bourgin et al., 2015; Marusak et al., 2015; Orem et al., 2019) in the context of the intergenerational transmission of stress (Thompson et al., 2011).

##### The Influence of rs*2740210* on Child Attachment and Disorganized Behavior

Here, we demonstrated that children carrying the risk allele (A) of the rs2740210 polymorphism show more disorganized behavior during the SSP. However, contrary to expectation, there was no evidence of an interaction with maternal CM. In line with our results, previous results indicated that the rs2740210 polymorphism was associated with externalizing psychiatric symptoms in children (Wade et al., 2016). However, the study only investigated children and therefore it could not be determined if the effect was moderated by early adverse maternal experiences. In a prospective study, it was shown that children carrying the C (risk) allele displayed stronger symptoms of general anxiety at the ages of 5–6 and emotional symptoms at the age of 11–12 when exposed to maternal verbal aggressive behavior in infancy (age 3 months; Smarius et al., 2020), indicating that the rs274021 polymorphism interacts with maternal behavior to predict the development of psychiatric symptoms in children, Yet, in our study, we could not find evidence for the interaction between early environmental stressors and the rs2740210 polymorphism on child disorganized behavior.

We suggest that future research should include biological factors with more attention since this information might be one explanatory factor for the heterogeneous findings reported in stress and attachment-related research. The physiological impact of rs2254298 and rs2740210 on the regulation of the oxytonergic system and its reactivity upon stress needs centered investigation. Here, the measurement of oxytocin, for example in saliva samples collected from mothers and children during the SSP could provide important biological insights.

The feasibility of such biologically centered approaches has already been demonstrated using the AAP (Krause et al., 2016). In addition to rs2254298 and rs2740210, also other targeted investigations with genetic candidates should be conducted. We emphasize here two other genes of physiological relevance to the stress and, most probably, to the attachment system. The first factor is the gene FKBP5 coding for the FK506 binding protein 5. FKBP5 interacts with the glucocorticoid receptor and influences the regulation and the sensitivity of the HPA-axis mediated stress response. SNPs within the FKBP5 gene were demonstrated to affect the cardiac stress reactivity in the context of early life adversity (Buchmann et al., 2014; Lovallo et al., 2016). The second HPA-associated factor in this context is NR3C1, the gene coding for the glucocorticoid receptor. Research that provided evidence for a genetic impact on the cardiac risk of individuals (Kuningas et al., 2006), further strengthen the necessity to co-assess SNPs within NR3C1 in the context of stress and attachment-focused research.

Finally, hypothesis-free approaches using genetic data could help to identify the physiological underpinnings of different attachment-based stress responses. Here, genome-wide association studies could be used. These studies require much larger samples sizes, which is a bottleneck for such a methodological approach.

#### Path Analysis of the Intergenerational Cycle of Maltreatment Model

Results of our path analysis confirmed results from the above-summarized literature: self-reported history of CM was associated with unresolved maternal attachment representation (all mothers with unresolved attachment representation have a history of CM). Moreover, and as expected, CM significantly affected the number of psychological symptoms, the degree of perceived social support in both our total cohort and subgroup of mothers with CM. Furthermore, CM influenced the degree of maternal perceived stress in the total cohort only. Maternal perceived stress had a significant influence on psychological symptoms. This implies that CM had an impact on mothers concerning several aspects relevant for caregiving within the intergenerational transmission of attachment and ICM. In this context, we found on a trend level, that especially in the subgroup of mothers exposed to CM, psychological symptoms affected maternal caregiving (disrupted behavior). However, this finding should be interpreted with caution.

The expected main attachment findings within our ICM model demonstrated that maternal attachment representation was highly associated with child attachment. On the other hand, maternal disruptive behavior was not related to child attachment but showed a trend toward significantly influencing the stress reactivity in children in the subgroup of mothers with CM. Interestingly, only in the CM+ group, the genetic factor rs2254298 OXTR genotype showed an effect on the cardiac stress response in children. This indicates, that in mother–child dyads with a higher maltreatment load (CM+) the genetic predisposition had an impact on the child’s autonomic nervous system reactivity during the SSP.

#### Concluding Remarks

The origins and transmission of a child history of maltreatment, independently from the severity of CM and its consequences on the child development, have been a focus in developmental and clinical research and intervention for over five decades. Since the Battered Child Syndrome was published by Helfer et al. (1997), psychologists and psychiatrists increasingly studied parenting factors that risk maltreating in the context of the intergenerational transmission of child abuse. Numerous studies in their original work and since that time have identified a list of factors that compound risk, including the studies reviewed here that included exploring maltreatment and impaired parenting in the context of the mechanisms of intergenerational transmission of attachment. Most studies, retrospective and prospective, have identified single factors or interacting factors as sources of distress and disruption in the family system and proposed intervention programs targeting these discoveries (Cyr et al., 2010).

The complexity and transdisciplinary approach in the present study examining the three systems –*attachment, support*, and *child biological susceptibility* – adds to this literature by identifying relevant *risk* and *protective* factors in the ICM model:

The results on the first hypothesis cluster on the *attachment system* all confirmed our expectations. CM was related to maternal attachment, especially in mothers with an unresolved attachment status. The maternal attachment was associated with child attachment suggesting that maternal unresolved attachment can be considered *a risk factor* and organized attachment a *protective factor* for child attachment. In contrast to our expectations, maternal caregiving (disruptive behavior) did not mediate between maternal attachment representation and child attachment. One explanation could be, that we have used another measure than in van IJzendoorn and Bakermans-Kranenburg (2019) model, who mostly reported on the impact of maternal sensitivity (Ainsworth, 1978) as one measure for caregiving quality. We preferred to use the AMBIANCE scale in this CM sample instead, in order to capture more relevant anomalous behaviors exhibited by caregivers in disorganized attachment relationships (Lyons-Ruth, 1996). However, we confirmed a significant positive association between maternal disruptive behavior and disorganized behavior of the child. Moreover, there was a significant association between maternal disruptive behavior and their child’s autonomic nervous system response. More specifically, more disruptive behavior was related to a higher increase in HR, and a steeper decline in RSA. In our cohort, maternal disruptive behavior was an expected *risk factor* for the child on a behavioral and biological level.

Our second hypothesis cluster examined mothers’ *social support systems*. As expected, we found that social support *attenuated* maternal problems. Greater amounts of perceived social support were significantly associated with reduced maternal stress and fewer psychological symptoms. The mediation analysis indicated that both social support and perceived stress mediated the effect of exposure to CM on the development of psychological symptoms later in life. We observed that a higher maltreatment load was associated with lower perceived social support and higher perceived stress levels, which increased the risk for the development of maternal psychopathology. This results pattern follows the intergenerational model of attachment by van IJzendoorn and Bakermans-Kranenburg (2019). However, we could not confirm a significant effect of perceived social support on maternal caregiving quality, which might be explained by the differential assessment of maternal caregiving quality. Moreover, counter to what had been expected, mothers with high disruptive behavior requested institutional support more frequently than mothers with low disruptive behavior.

Our examination of the genetic variability within the OXTR gene (rs2254298) revealed a significant influence on the cardiac stress response of children from CM+ mothers; however, this observation could not be replicated in the total sample. Based on our results, we confirm a gene × environment interaction of maternal CM and the rs2254298 polymorphism and showed that the A (risk) allele is a *risk factor* for the cardiac stress response in the context of ICM. In addition, for the rs2740210 polymorphism, we could show that children carrying the C (risk) allele displayed more disorganized behavior during the SSP, independent of maternal CM exposure.

In sum, the current study combined a multi-systems approach to understanding risk and buffering factors that demonstrate resilience in mothers who have experienced CM and factors that can interrupt developmental risk in their children. We followed a two-step approach in the analyses of this manuscript. First, we intended to replicate core findings from the literature regarding intergenerational paths in the transmission of CM. Here, we recruited a cohort of women who very recently became mothers from the population of Ulm. Various effects might have led to discrepancies between results from single analyses vs. path analysis. For example, the availability and consistency of data (sets) used in the analyses slightly differ, leading to different results. We want to stimulate more research in this field also using our extended path to test the robustness of our initial findings reported here as well as by adding additional future variables that might help to better understand and disentangle intergenerational transmission of CM.

Our study has several strengths. First, the high level of interdisciplinarity used to cover the complexity in the ICM and attachment included not only psychological and psychosocial factors but also cardiovascular and genetic features. Second, the prospective and longitudinal study design enabled us to combine mother–child dyadic observations from several time points starting from parturition extending for 12 months. Third, we empirically tested the model by van IJzendoorn and Bakermans-Kranenburg (2019) and extended for the first time that model to include cardiovascular and genetic features related to the oxytocinergic system as important risk and resilience factors that could be relevant for improved interventions for preventing ICM.

Our results are constrained by several limitations including the relatively small sample size. Non-significant results might be the results of the relatively small number of participants. The mothers in our study also had a relatively low CM load, limiting the general validity of our findings, highlighting the importance of replication in larger cohorts with more pronounced CM load, for example in clinical cohorts.

The current findings further advance our understanding of risk and vulnerability factors and enable professionals to target and offer adequate services to parents and children at risk. We would like to highlight the role of the OXTR as an important mediator of stress transmission from mother to child. Future studies should explore the reactivity of the oxytocinergic system in mother–child dyads in the context of maternal attachment and ICM. The consideration of genetic risk and resilience factors in future work could open the gate toward personalized and therefore more tailored treatment approaches. The identification of more vulnerable groups based on OXT(R) genotypes could help to implement more tailored clinical interventions. For example, pharmacological treatments targeting the oxytocinergic system might improve stress-regulatory function. One rather neglected feature in the context of ICM includes the role of paternal attachment as a potential risk or resilience factor and should be co-assessed in future studies.

## Data Availability Statement

The original contributions presented in this study are included in the article/Supplementary Material, further inquiries can be directed to the corresponding author.

## Ethics Statement

The studies involving human participants were reviewed and approved by Local Ethics Board at Ulm University. Written informed consent to participate in this study was provided by the participants’ legal guardian/next of kin.

## Author Contributions

JF, UZ, CW, HK, AB, and HG conceptualized the design of the study. UZ had the lead for the assessment of the child attachment data and mother-child interactions. CW for the child cardiovascular assessments. AK for the assessment and analyses of the genetic data. HG and AB for the maternal data. AB for the assessment of the maternal attachment interviews (AAP), rated the AAP interviews together with a certified judge, and wrote the manuscript with contributions from all co-authors. UZ and AB were responsible for organizing the conduction of the statistical analyses by external experts, see section “Acknowledgments”. AB, UZ, and HK conceptualized the manuscript design. All authors approved the final version of the manuscript.

## Conflict of Interest

The authors declare that the research was conducted in the absence of any commercial or financial relationships that could be construed as a potential conflict of interest.

## Publisher’s Note

All claims expressed in this article are solely those of the authors and do not necessarily represent those of their affiliated organizations, or those of the publisher, the editors and the reviewers. Any product that may be evaluated in this article, or claim that may be made by its manufacturer, is not guaranteed or endorsed by the publisher.
