# Supplementary material for: Identifying Risk and Resilience Factors in the Intergenerational Cycle of Maltreatment: Results From the TRANS-GEN Study Investigating the Effects of Maternal Attachment and Social Support on Child Attachment and Cardiovascular Stress Physiology
Source: Front Hum Neurosci. 2022 Jul 18;16:890262. doi: 10.3389/fnhum.2022.890262 (PMC9341217; doi:10.3389/fnhum.2022.890262)
Supplement: Supplementary file 1 [file Table_1.docx]

**Table S1**. Correlations among the study variables (CM+ and CM- group).

| **Variables** |  | 1 | 2 | 3 | 4 | 5 | 6 | 7 | 8 | 9 | 10 | 11 | 12 | 13 |  |
| --- | --- | --- | --- | --- | --- | --- | --- | --- | --- | --- | --- | --- | --- | --- | --- |
| 1. CM (CTQ) | *r* | - |  |  |  |  |  |  |  |  |  |  |  |  |  |
|  | *p* | - |  |  |  |  |  |  |  |  |  |  |  |  |  |
| 2. Maternal attachment (AAP) | *r* | .35*** | - |  |  |  |  |  |  |  |  |  |  |  |  |
|  | *p* | **< .001** | - |  |  |  |  |  |  |  |  |  |  |  |  |
| 3. Maternal caregiving (Ambiance) | *r* | .12 | .15^†^ | - |  |  |  |  |  |  |  |  |  |  |  |
|  | *p* | .14 | .06 | - |  |  |  |  |  |  |  |  |  |  |  |
| 4. Perceived stress (PSS14) | *r* | .31*** | .10 | .07 | - |  |  |  |  |  |  |  |  |  |  |
|  | *P* | **< .001** | .21 | .41 | - |  |  |  |  |  |  |  |  |  |  |
| 5. Psychological symptoms (BSI) | *r* | .48*** | .16* | .14^†^ | .73*** | - |  |  |  |  |  |  |  |  |  |
|  | *p* | **< .001** | **.04** | .07 | **< .001** | - |  |  |  |  |  |  |  |  |  |
| 6. Social support (PSSQ) | *r* | -.42*** | -.20* | -.13 | -.31*** | -.38*** | - |  |  |  |  |  |  |  |  |
|  | *p* | **< .001** | **.012** | .12 | **< .001** | **< .001** | - |  |  |  |  |  |  |  |  |
| 7. Institutional support | *r* | .09 | .10 | .20* | .07 | .13 | -.01 | - |  |  |  |  |  |  |  |
|  | *p* | .39 | .32 | **.04** | .46 | .19 | .92 | - |  |  |  |  |  |  |  |
| 8. rs2254298 genotype | *r* | .02 | .16 | .06 | -.01 | -.11 | .05 | -.05 | - |  |  |  |  |  |  |
|  | *p* | .86 | .10 | .58 | .90 | .27 | .61 | .67 | - |  |  |  |  |  |  |
| 9. rs2740210 genotype | *r* | -.03 | .07 | .22* | .07 | .03 | .12 | .19 | .05 | - |  |  |  |  |  |
|  | *p* | .78 | .51 | **.03** | .50 | .74 | .24 | .13 | .62 | - |  |  |  |  |  |
| 10. Child stress response (HR%) | *r* | -.04 | -.11 | .17* | -.05 | -.05 | .15^†^ | .07 | -.01 | -.07^†^ | - |  |  |  |  |
|  | *p* | .66 | .18 | **.04** | .50 | .57 | .07 | .52 | .93 | .47 | - |  |  |  |  |
| 11. Child Stress response (RSA%) | *r* | -.03 | -.02 | -.17* | -.003 | .03 | -.10 | -.21* | -.10 | -.11 | -.68*** | - |  |  |  |
|  | *p* | .72 | .80 | **.04** | .97 | .75 | .22 | **.03** | .33 | .29 | **< .001** | - |  |  |  |
| 12. Child attachment (FST) | *r* | .18* | .26** | .14^†^ | -.10 | -.001 | -.11 | .09 | .02 | .18 | -.07 | .04 | - |  |  |
|  | *p* | **.03** | **.002** | .10 | .21 | .99 | .19 | .39 | .84 | .09 | .43 | .68 | - |  |  |
| 13. D-score | *r* | .07 | .08 | .26** | -.06 | .11 | .03 | .13 | -.19 | .27* | -.09 | .07 | .68*** | - |  |
|  | *p* | .48 | .40 | **.008** | .55 | .28 | .73 | .28 | .11 | **.02** | .39 | .48 | **< .001** | - |  |
|  |  |  |  |  |  |  |  |  |  |  |  |  |  |  |  |

Note, ****p* < .001, ***p* < .01, **p* < .05, ^†^*p* < .10 (marginal significance). Significant results are given in bold.
