# Supplementary material for: Identifying Risk and Resilience Factors in the Intergenerational Cycle of Maltreatment: Results From the TRANS-GEN Study Investigating the Effects of Maternal Attachment and Social Support on Child Attachment and Cardiovascular Stress Physiology
Source: Front Hum Neurosci. 2022 Jul 18;16:890262. doi: 10.3389/fnhum.2022.890262 (PMC9341217; doi:10.3389/fnhum.2022.890262)
Supplement: Supplementary file 2 [file Table_2.docx]

**Table S2**. Correlations among the study variables in the CM+ group

| **Variables** |  | 1 | 2 | 3 | 4 | 5 | 6 | 7 | 8 | 9 | 10 | 11 | 12 | 13 |
| --- | --- | --- | --- | --- | --- | --- | --- | --- | --- | --- | --- | --- | --- | --- |
| 1. CM (CTQ) | *r* | - |  |  |  |  |  |  |  |  |  |  |  |  |
|  | *p* | - |  |  |  |  |  |  |  |  |  |  |  |  |
| 2. Maternal attachment (AAP) | *r* | .30* | - |  |  |  |  |  |  |  |  |  |  |  |
|  | *p* | **.01** | - |  |  |  |  |  |  |  |  |  |  |  |
| 3. Maternal caregiving (Ambiance) | *r* | .08 | .14 | - |  |  |  |  |  |  |  |  |  |  |
|  | *p* | .52 | .26 | - |  |  |  |  |  |  |  |  |  |  |
| 4. Perceived stress (PSS14) | *r* | .15 | .04 | .14 | - |  |  |  |  |  |  |  |  |  |
|  | *p* | .22 | .74 | .25 | - |  |  |  |  |  |  |  |  |  |
| 5. Psychological symptoms (BSI) | *r* | .37** | .06 | .20^†^ | .70*** | - |  |  |  |  |  |  |  |  |
|  | *p* | **.001** | .62 | .099 | **< .001** | - |  |  |  |  |  |  |  |  |
| 6. Social support (PSSQ) | *r* | -.27* | -.11 | -.19 | -.17 | -.24* | - |  |  |  |  |  |  |  |
|  | *p* | **.02** | .37 | .11 | .16 | **.047** | - |  |  |  |  |  |  |  |
| 7. Institutional support | *r* | -.04 | .09 | .15 | .07 | .12 | -.12 | - |  |  |  |  |  |  |
|  | *p* | .81 | .55 | .34 | .64 | .44 | .46 | - |  |  |  |  |  |  |
| 8. rs2254298 genotype | *r* | .20 | .31 | .15 | .15 | .19 | .03 | .12 | - |  |  |  |  |  |
|  | *p* | .19 | **.04** | .35 | .34 | .22 | .86 | .56 | - |  |  |  |  |  |
| 9. rs2740210 genotype | *r* | .26 | .21 | .36* | .05 | .08 | .16 | .01 | .09 | - |  |  |  |  |
|  | *p* | .09 | .17 | **.02** | .77 | .61 | .32 | .96 | .57 | - |  |  |  |  |
| 10. Child stress response (HR%) | *r* | .07 | -.15 | .13 | .14 | .10 | .02 | .04 | .34* | -.18 | - |  |  |  |
|  | *p* | .59 | .21 | .30 | .26 | .40 | .87 | .79 | **.02** | .25 | - |  |  |  |
| 11. Child stress response (RSA%) | *r* | -.21^†^ | -.06 | -.27* | -.16 | -.12 | .03 | -.37* | -.47** | -.25 | -.58 | - |  |  |
|  | *p* | .08 | .61 | **.03** | .20 | .32 | .84 | **.02** | **.002** | .12 | **< .001** | - |  |  |
| 12. Child attachment (FST) | *r* | .16* | .30* | .12 | -.18 | -.09 | -.01 | .05 | -.11 | .24 | -.18 | .08 | - |  |
|  | *p* | .21 | **.02** | .34 | .15 | .47 | .93 | .76 | .51 | .12 | .15 | .53 | - |  |
| 13. D-score | *r* | -.004 | .07 | .31* | -.03 | .14 | .09 | .20 | -.22 | .27 | -.28 | .09 | .72*** | - |
|  | *p* | .98 | .61 | **.03** | .82 | .33 | .53 | .25 | .23 | .13 | **.05** | .55 | **< .001** | - |
|  |  |  |  |  |  |  |  |  |  |  |  |  |  |  |

Note, ****p* < .001, ***p* < .01, **p* < .05, ^†^*p* < .10 (marginal significance). Significant results are given in bold.
